# Exosome-Enriched Plasma Analysis as a Tool for the Early Detection of Hypertensive Gestations

**DOI:** 10.3389/fphys.2021.767112

**Published:** 2021-12-14

**Authors:** Rodrigo Barbano Weingrill, Sandra Luft Paladino, Matheus Leite Ramos Souza, Eduardo Manoel Pereira, Aldilane Lays Xavier Marques, Elaine Cristina Oliveira Silva, Eduardo Jorge da Silva Fonseca, Jeferson Santana Ursulino, Thiago Mendonça Aquino, Estela Bevilacqua, Johann Urschitz, Jean Carl Silva, Alexandre Urban Borbely

**Affiliations:** ^1^Programa de Pós-Graduação em Saúde e Meio Ambiente, Universidade da Região de Joinville – UNIVILLE, Joinville, Brazil; ^2^Institute for Biogenesis Research, John A. Burns School of Medicine, University of Hawai‘i at Mānoa, Honolulu, HI, United States; ^3^High Risky Gestation Ambulatory, Darcy Vargas Maternity, Joinville, Brazil; ^4^Cell Biology Laboratory, Institute of Health and Biological Sciences, Federal University of Alagoas, Maceió, Brazil; ^5^Optics and Nanoscopy Group, Institute of Physics, Federal University of Alagoas, Maceió, Brazil; ^6^Nucleus of Analysis and Research in Nuclear Magnetic Resonance, Institute of Chemistry and Biotechnology, Federal University of Alagoas, Maceió, Brazil; ^7^Laboratory for Maternal-Fetal Interactions and Placenta Research, Department of Cellular and Developmental Biology, Institute of Biomedical Sciences, University of São Paulo, São Paulo, Brazil

**Keywords:** Raman spectroscopy, hypertensive gestational disorders, exosome-enriched plasma, ^1^H NMR, biomarkers

## Abstract

Hypertensive disorders of pregnancy are closely associated with prematurity, stillbirth, and maternal morbidity and mortality. The onset of hypertensive disorders of pregnancy (HDP) is generally noticed after the 20th week of gestation, limiting earlier intervention. The placenta is directly responsible for modulating local and systemic physiology by communicating using mechanisms such as the release of extracellular vesicles, especially exosomes. In this study, we postulated that an analysis of exosome-enriched maternal plasma could provide a more focused and applicable approach for diagnosing HDP earlier in pregnancy. Therefore, the peripheral blood plasma of 24 pregnant women (11 controls, 13 HDP) was collected between 20th and 24th gestational weeks and centrifuged for exosome enrichment. Exosome-enriched plasma samples were analyzed by Raman spectroscopy and by proton nuclear magnetic resonance metabolomics (^1^H NMR). Principal component analysis (PCA) and orthogonal partial least squares discriminant analysis (OPLS-DA) were used to analyze the Raman data, from the spectral region of 600–1,800 cm^–1^, to determine its potential to discriminate between groups. Using principal component analysis, we were able to differentiate the two groups, with 89% of all variances found in the first three principal components. In patients with HDP, most significant differences in Raman bands intensity were found for sphingomyelin, acetyl CoA, methionine, DNA, RNA, phenylalanine, tryptophan, carotenoids, tyrosine, arginine, leucine, amide I and III, and phospholipids. The ^1^H NMR analysis showed reduced levels of D-glucose, L-proline, L-tyrosine, glycine, and anserine in HDP, while levels of 2-hydroxyvalerate, polyunsaturated fatty acids, and very-low-density lipoprotein (VLDL) were increased. ^1^H NMR results were able to assign an unknown sample to either the control or HDP groups at a precision of 88.3% using orthogonal partial least squares discriminant analysis and 87% using logistic regression analysis. Our results suggested that an analysis of exosome-enriched plasma could provide an initial assessment of placental function at the maternal-fetal interface and aid HDP diagnosis, prognosis, and treatment, as well as to detect novel, early biomarkers for HDP.

## Introduction

Hypertensive disorders of pregnancy affected more than 18 million pregnancies worldwide in 2019 (Wang et al., 2021). This condition is one of the leading causes of maternal and fetal morbidity and mortality, and a critical threat to maternal and infant health (Garovic et al., 2020). In 2013, the American Congress of Obstetricians and Gynecologists (ACOG) classified hypertensive disorders of pregnancy (HDP) into four categories: gestational hypertension, preeclampsia/eclampsia, chronic hypertension, and chronic hypertension complicated with preeclampsia/eclampsia (American College of Obstetricians and Gynecologists (ACOG), 2019). In 2020, the International Society of Hypertension guidelines maintained the four categories, although separately categorizing the hemolytic anemia, elevated liver enzymes, and low platelet count syndrome (HELLP syndrome) (Unger et al., 2020). HDP risk assessment and prevention has helped to reduce maternal mortality in Europe and North America, although rates are still very high (Abalos et al., 2013). HDP is even more prevalent in Latin American countries, with Brazil and Mexico having the highest incidence rates (Wang et al., 2021). Regarding incidence trends, an increase was observed mainly in Andean Latin America and the Caribbean in 2019, with Ecuador showing one of the greatest increasing trends (Wang et al., 2021). Therefore, considering the relevance of HDP, non-invasive early diagnosis and prognosis management are essential, particularly in Latin American countries.

It is believed that the placenta plays a significant role in HDP establishment, as both ischemia and hypoxia of the placenta elicit an adverse maternal circulatory milieu, leading to excessive production and release of pro-inflammatory cytokines, defective angiogenic factors, and reactive oxygen species (Parrish et al., 2011). These bioactive factors target blood vessels, resulting in vascular smooth cell hypertrophy and endothelial dysfunction, which eventually lead to vasoconstriction, increased peripheral vascular resistance, and HDP (Myatt and Webster, 2009). In this context, small extracellular vesicles (exosomes) produced by the placenta are thought to play a key role in HDP. Previous studies have shown that pregnant women with HDP have a higher placental to total exosome ratio compared to controls, which may play a negative role in angiogenesis due to unbalanced delivery of proangiogenic, antiangiogenic, and immunological factors (Pillay et al., 2017; Czernek and Düchler, 2020). More importantly, it is suggested that placental exosomes are responsible for the onset of HDP due to defected endothelial remodeling signaling *via* microRNA cargo, as well as caring elevated levels of soluble fms-like tyrosine kinase-1 (t sFlt-1) and soluble endoglin (sEng), both known to be a causative agent of pre-eclampsia (Pillay et al., 2019; Hashimoto et al., 2021). Moreover, placental exosomes are also considered as biomarkers of preeclampsia development, since the release of these structures to the maternal circulation occurs especially in the first half of pregnancy (Tong and Chamley, 2015; Li et al., 2019), and their isolation and characterization can be a tool to study the differences found in healthy and pathological placentas (Pillay et al., 2017; Burkova et al., 2021).

Among several techniques that can be used to characterize and diagnose HDP using plasma samples, Raman spectroscopy and proton nuclear magnetic resonance metabolomics (^1^H NMR) also has the potential to detect novel biomarkers. Both techniques are commonly used for diagnosis and prognosis for a wide range of diseases (Kong et al., 2015; Wishart, 2019). Raman spectroscopy identifies differences in the biochemical signature, where slight changes in chemical interactions can be related to proteins, RNAs, carbohydrates, and lipids, while other techniques are usually limited to a particular class of molecules (Kong et al., 2015). Moreover, while transcriptomics and proteomics tell us what can happen in organisms, metabolomics gives us information on organisms’ current state and what happened to them (Wishart, 2019). Hence, in this study, we aimed to expand the use of exosomes with the analysis of how exosome-enriched maternal plasma could provide a focused and applicable approach on HDP diagnosis using Raman spectroscopy and proton nuclear magnetic resonance metabolomics (^1^H NMR).

## Materials and Methods

### Samples Collection and Storage

Subjects were selected at the High-Risk Outpatient Clinic of the Darcy Vargas Maternity, Joinville, Santa Catarina, Brazil, from July to December 2019. The study obtained approval from the Ethics Committee (3.276.544/2019) where ethical considerations were based on the use of the material for scientific purposes, with confidentiality of the subject identity and without constraint from the institutions or people involved. Routinely, patients at the satellite health centers were admitted to the High-Risk Clinic after presenting the following altered parameters: at least three measures of Diastolic ≥ 140/90 mmHg, protein/creatinine ratio, fasting blood glucose ≥ 92 mg/dl; TTOG ≥ 153 mg/dl. For HDP, only subjects with at least three measures of Diastolic ≥ 140/90 mmHg and protein/creatinine ratio alterations were selected, where obesity, gestational diabetes, or other gestational disorders were reasons for exclusion. The control group was composed of subjects that had no clinical or laboratory diagnostic abnormalities. Subjects were monitored until the end of their gestations to confirm that they had no further pregnancy complications.

### Peripheral Blood Collection

Peripheral blood was collected on heparin-coated tubes (BD Vacutainer, United States) from 24 pregnant women (11 controls, 13 HDP) between 20th and 24th gestational weeks. Pregnant women from 18 to 35 of age, with no current gestational complications (other than HDP), autoimmune and infectious diseases, or genetic disorders were recruited. The subjects who decided to not continue participation in this study or who did not show up for follow-up of prenatal care at the referred service, subjects who used tobacco and/or alcohol during pregnancy were dropped from the analysis.

### Enrichment of Plasma With Small Extracellular Vesicles (Exosomes)

To separate blood cells and plasma, peripheral blood was centrifuged immediately after collection at 1,500 rpm at 4°C for 15 min. To enrich plasma with small extracellular vesicles, samples were differentially centrifuged at 1,200 *g* for 20 min at 4°C to eliminate large extracellular vesicles (apoptotic bodies), cells, and membrane debris. The supernatants were carefully collected and centrifuged at 10,000 *g* for 30 min at 4°C to precipitate large extracellular vesicles. Again, supernatant, representing exosome enriched plasma was carefully collected and immediately stored at –80°C. For thawing, samples were submerged in a water bath at 37°C (Bunggulawa et al., 2018; Théry et al., 2018; Liu et al., 2020).

### Nanoparticle Tracking Analysis of Exosome Enriched Plasma

For the determination of enriched plasma particle concentration and size distribution, nanoparticle tracking analysis (NTA) was performed using a Nanosight NS300 (Malvern Instruments Ltd., Malvern, United Kingdom), equipped with a 488 nm blue laser. Samples were previously diluted on double-filtered PBS buffer to reach optimal instrument concentration while avoiding particle contamination. Readings were made at 25 frames/s, camera level set to 14, and temperature of 25°C. Particle analysis was performed using the NTA Software version 3.4 build 3.4.003 (Malvern Panalytical, Malvern, United Kingdom). Particle concentration, average size (Mean), and most abundant size on the sample (Mode) were evaluated to validate sample particle size.

### Raman Spectroscopy

The Raman spectra were measured using an XploRA spectrometer (Horiba, Japan) coupled to an optical microscope (BXFM, Olympus, Japan) and equipped with a 532 nm laser that was focused on the samples through a 100 × objective (NA = 0.9). The same objective lens was used for collecting Raman scattered light after interaction with the sample, in backscattering geometry. The frequency calibration was set by reference to the 520 cm^–1^ vibrational bands of a silicon wafer. Under the same conditions, five Raman spectra were captured from each sample of both groups in the spectral range of 600–1,800 cm^–1^. To minimize laser-induced heating of the specimens, low power irradiation at the sample surface was used, around 5 mW, combined with a short exposure time (3 s laser exposure for five accumulations). The diffraction grating used had 1,200 lines/mm, which yielded a spectral resolution of 1.5 cm^–1^.

### Raman Spectroscopy Data Preprocessing and Spectral Analysis

Before conducting the spectral analysis, all spectra were smoothed, background-adjusted, and normalized by the area using an algorithm implemented in MatLab software (Mathworks, Naticks, MA, United States). As such, the external noises were suppressed and useful information about the biochemical composition was enhanced. After removing the fluorescence background from the spectra, principal component analysis (PCA) was performed to evaluate the spectral variability in the dataset through an algorithm implemented in Matlab software. Orthogonal partial least squares discriminant analysis (OPLS-DA) was performed to predict which class each sample belonged to, using the SIMCA software (Sartorius AG, Göttingen, Germany). All multivariate statistical analyses were implemented in MatLab software. Raman individual bands with statistically significant fluctuations were isolated for further comparison. From the most significant changes in PCA, the Raman bands were assigned as well as the main contributors to them (Table 1). The interpretation of the spectral features was based on the published literature (Gelder et al., 2007; Movasaghi et al., 2007; Başar et al., 2012; Czamara et al., 2015; Surmacki et al., 2015; Talari et al., 2015; Kelestemur and Çulha, 2016; Nguyen et al., 2017; Balan et al., 2019; Bai et al., 2020).

**TABLE 1:** Raman bands position, their respective assignments and contributors.

Raman band position (cm^–1^)	Band assignment	Contributions
629	β(C-C)	Uric acid
724	βCH	Acetyl-CoA, CoA, Methionine and Sphingomyelin
966	υ(C-C)	DNA backbone and RNA
1,001	Ring breathing modes	Phenylalanine
1,012	Strong Van der Walls force interactions	Tryptophan
1,020	υ(C = O)	*N*-acetyl-D-glucosamine
1,152	υ(C-C)	Carotenoids
1,164	Ring breathing modes	Tyrosine and Arginine
1,175	βCH	Tyrosine, Leucine and Phenylalanine
1,201	υCH	Tyrosine
1,291	βCH	Amide III (α-helix)
1,456	υCH_2_, υ_a_CH_3_	Proteins and Phospholipids
1,507	υ_a_(C-C)	Myristic acid
1,514	υ(C = C)	Carotenoids
1,517	υ(C-C)	Carotenoids (β-carotene)
1,525	υ(C = C)	Carotenoids
1,535	υ(C = C)	Carotenoids (β-carotene)
1,668	υ(C = C)	Amide I (β-sheet)

*β- bending, υ-stretching, υ_a_-asymmetric stretching (Gelder et al., 2007; Movasaghi et al., 2007; Başar et al., 2012; Czamara et al., 2015; Surmacki et al., 2015; Talari et al., 2015; Kelestemur and Çulha, 2016; Nguyen et al., 2017; Balan et al., 2019; Bai et al., 2020).*

### Statistical Analysis

To examine if all data were normally distributed, a Kolmogorov-Smirnov test was performed. Matlab software was used for spectral analysis, and PCA and OPLS-DA were used for multivariate statistical analyses. Other results were analyzed using Graph Pad Prism (Graph Pad Software Inc., San Diego, CA, United States) with a two-tailed Mann–Whitney test. The minimal level of significance for all experiments was set at *p* < 0.05. The results are depicted as the mean ± SEM.

### Proton Nuclear Magnetic Resonance Metabolomics Calibration and Readings

The exosomes-enriched plasma samples were prepared by the addition of 350 μl phosphate buffer (containing trimethylsilylpropionic acid-d4 sodium salt, TSP.1 mM, and 100% D_2_O), with further transference to 5 mm NMR tubes. All ^1^H NMR spectra were acquired on a Bruker 600 MHz spectrometer Avance III (Bruker BioSpin, Germany) equipped with a probe PA BBO 600 S3 BBF-H-D-05 Z SP, operated at a 1 H frequency of 600.13 MHz. Before starting the experiments, the NMR spectrometer was calibrated daily following strict standard operation procedures to ensure the highest spectral quality and reproducibility. Every morning, NMR standard reference samples (5 mm probe) methanol-d4 (MeOD) and 2 mM sucrose were acquired to check the spectrometer’s optimal conditions (Findeisen et al., 2007; Bruzzone et al., 2020). Additionally, we checked a stable temperature in the NMR probe to prevent variations of chemical shifts, particularly the water peak for water suppression. For this purpose, a calibration sealed tube containing a 99.8% MeOD standard sample was used to ensure that plasma samples were run at 298 K. The stability of temperature over time in the NMR probe was controlled with the command “edte.” The second calibration tube was a sucrose sample to optimize the water suppression and spectrometer sensitivity. An automatic tune and matching were performed, locked (90% H2O + 10% D2O_salt), and automatically shimmed (topshim 3D 1H ordmax = 8). ^1^H NMR spectra were recorded using pulse sequence Carr-Purcell-Meiboom-Gill (CPMG) with water presaturation (pulse program: cpmgpr1d), implementing a T2 filter to suppress the broad signals of proteins and other macromolecules. This NMR sequence allows the selective detection of signals arising only from low molecular weight metabolites. For all experiments, 128 scans were recorded after 16 dummy scans, 64k data points, a spectral width of 20.029 ppm, relaxation delay of 4 s, 2.73 s acquisition time, and mixing time of 10 ms. Before applying the Fourier transform, free induction decays were multiplied by an exponential function equivalent to a 0.3 Hz line-broadening factor. The processing spectral was realized automatically correcting phase and baseline and calibrated to the TSP at δ0.00 ppm, using TopSpin 3.5 (Bruker BioSpin, Billerica, MS, United States). The peaks were identified in the one-dimensional spectra using The Human Metabolome Database (HMDB) (Wishart et al., 2018) version 4 (The Metabolomics Innovation Centre, Canada), and the ChenomX NMR Suite (Chenomx Inc., Canada).

### ^1^H NMR Data Processing and Statistical Analysis

After automatic processing spectral by TopSpin, the dataset was processed using the R statistical software, version 3.6^1^ with R package mrbin version 1.5 (R Core Team, Vienna, Austria). The ^1^H NMR spectrum dataset was aligned and bucketed into consecutive bins of fixed 0.03 ppm width over the region from 0.5 to 9.5 ppm. The spectral region between 4.7 and 5.1 ppm (containing the residual water signal) was excluded. The online software MetaboAnalyst version 5 (Wishart Research Group, University of Alberta, Canada) was employed for biomarker discovery and classification (Chong et al., 2019). All bins respective intensities were normalized by a median to minimize the effect of different concentrations. Unsupervised PCA was used as the first exploratory analysis to obtain a preliminary data outlook and facilitate a general overview over both groups (control and HDP) *via* the principal components (PC1 vs. PC2) in score plots, trends revealing, or potentials outliers. At the same time, the loadings plot provided insight into the weights of variables in each component. In addition, Heatmap analysis showing clustering metabolic patterns between the control and HDP was applied. The model OPLS-DA was performed to assess metabolomics data’s capacity in distinguishing the HDP from control samples for the complete set of metabolites identified. The defined confidence region was 95%, and several permutations were set to 2,000 for model evaluation. Significant metabolites (biomarkers) were selected by the Loading S-plot from the OPLS-DA showing the importance of variables in the model, combining the covariance and the correlation (p(corr)) loading profile.

## Results

### General Characteristics of the Study Population

The general characteristics are depicted in Table 2. No relevant social demographic differences were found, nor were differences in the prevalence of HDP according to age, skin color, family history of HDP, and several previous pregnancies. The average gestational age of the control group was 38.3 weeks, whereas HDP pregnancies ended at an average of 36 weeks (*p* < 0.05).

**TABLE 2:** General characteristics between normal vs. subjects with Hypertensive Disorders in Pregnancy (HDP) (≥ 140/90 mmHg).

	Control (*n* = 11)	HDP (*n* = 13)
Age	28.4 ± 7.5	29.8 ± 4.9
HDP onset (weeks ± days)	-	25 ± 6.2
**N° of Previous Pregnancies**		
0	5 (45.45%)	2 (15.38%)
1	2 (18.18)	9 (69.24%)
2	3 (27.27)	1 (7.69%)
3	1 (9.10%)	1 (7.69%)
Family history of HDP	7 (63%)	7 (53%)
Vaginal delivery	2 (18.18%)	1 (7.69%)
Caesarian section	5 (45.45%)	11 (84.62%)
Delivery type not informed	4 (36.37%)	1 (7.69%)
Gestational age at delivery	38.3 ± 0.9 weeks	36.0 ± 0.7 weeks*

*Statistically significant (P < 0.05).

### Enriched Plasma Nanoparticle Tracking Analysis

**FIGURE 1:**
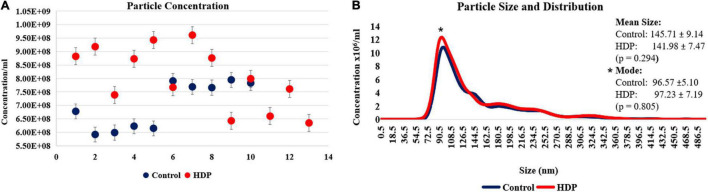
Nanoparticle tracking analysis of enriched plasma. **(A)** Overall nanoparticle tracking analysis (NTA) of enriched plasma shows an increase in particle concentration on HDP (8.04 ± 1.14 × 10^8^ nm; *n* = 13) when compared with controls (7.01 ± 0.87 × 10^8^ nm; *n* = 10; *p* = 0.027). **(B)** Particle size distribution revels no significant differences on Mean and Mode particle concentration on HDP and control (Mean of 141.98 ± 7.47 nm, and 145.71 ± 9.14 nm; Mode of 97.23 ± 7.19 nm, and 96.57 ± 5.10 nm, respectively; *p* = 0.805).

Through NTA analysis, particle concentration and particle size distribution on control and HDP samples were accessed. HDP enriched plasma displayed higher overall particle concentration when compared with controls (8.04 ± 1.14 × 10^8^/ml and 7.01 ± 0.87 × 10^8^/ml, respectively; *p* = 0.027) (Figure 1A). Particle size distribution analysis of average size particles (mean) and the most abundant particles of each sample (mode) showed no significant differences between controls and HDP (mean size 145.71 ± 9.14 and 141.98 ± 7.47 nm, respectively; and mode: 96.57 ± 5.1 and 97.23 ± 7.19 nm, respectively) (Figure 1B). In this context, the difference in overall particle concentration does not reflect any sort of large particles contamination. The enriched plasma samples indeed contained mostly small extracellular vesicles based on size distribution (50–150 nm; mean and mode; Figure 1).

### Raman Spectra Classification and Analysis

**FIGURE 2:**
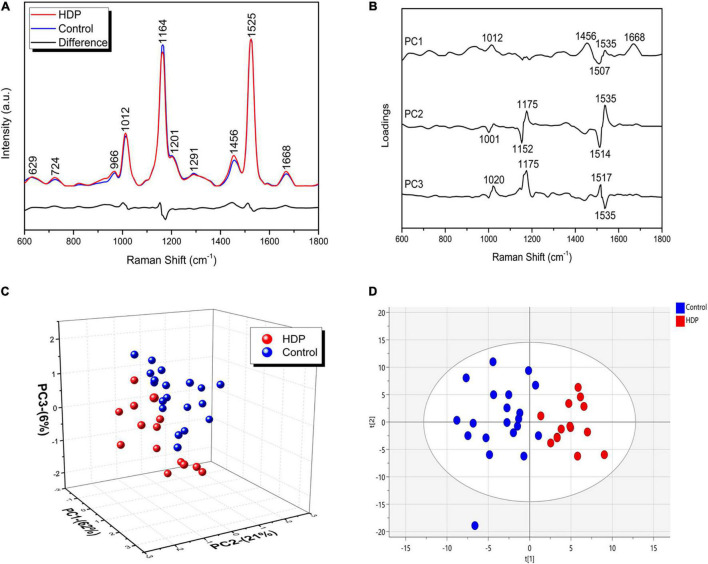
HDP changes the exosome-enriched plasma biochemical signature. **(A)** The Raman spectra represent the averages of five spectra captured from each sample of both groups (Control and HDP) in the fingerprint region (600–1,800 cm^–1^). Main Raman spectra from control and HDP with the most variable bands; **(B)** Loadings of PC1, PC2, and PC3 for Control and HDP; **(C)** Three-dimensional PCA score plot. **(D)** Two-dimensional OPLS-DA score plot of the Raman spectra for the control and HDP groups. The two groups were relatively well discriminated along t[1] axis, as well along t[2] axis.

Through the Raman spectra, it was possible to identify the most important spectral differences between control and HDP groups. A total of five spectra were acquired from each patient, and their average Raman spectra and the most variable areas are depicted in Figure 2A. Herein, principal component analysis (PCA) was used for the classification and interpretation of the spectral data. A 3D plot was constructed with combinations of sets of scores of the first three PCs as well as the corresponding plots of the PC1, PC2, and PC3 loadings, used for the determination of the differentiation capability of PCA and identification of significant Raman features (Figures 2B,C). The PC loadings are representative of the biochemical differences between the groups and are responsible for the differentiation of the spectra in the score plot of PC. In the PCA, loadings of PC1 indicated positive correlations of Raman bands at 1,012, 1,456, 1,535, and 1,668 cm^–1^, and negative correlations at 1,507 cm^–1^. The PC2 loadings indicated positive correlations at 1,175 and 1,535 cm^–1^, whereas negative correlations at 1,001, 1,152, and 1,514 cm^–1^. The PC3 loadings indicated positive correlations at 1,020, 1,175, and 1,517 cm^–1^, whereas negative correlation at 1,535 cm^–1^ (Figure 2B). As such, the first three PC explained 89% of the variance of the original data set, with PC1 describing 62%, PC2 describing 21%, and PC3 describing 8% of the total variance, with PCA evidently dividing the two groups into two different clusters, indicating spectral differences between the analyzed groups (Figure 2C). Supervised pattern recognition techniques use sample class information to build classification models capable of predicting which class a particular sample belongs to. Herein, a supervised form of discriminant analysis (OPLS-DA) was also applied to the results to highlight the differences between the groups. Figure 2D shows that the two types of samples were separated from each other completely. OPLS-DA of the Raman spectra resulted in the following specific parameters: R^2^X(cum) = 0.834, R^2^Y(cum) = 0.953, and Q^2^(cum) = 0.889 (*p* < 0.05). Control and HDP groups were relatively well discriminated along the X-axis using this method, as well along the Y-axis. The three parameters above were all > 0.7, thereby indicating that the model was well established and presented a good prediction ability.

### Raman Spectra Assignment and Interpretation

**FIGURE 3:**
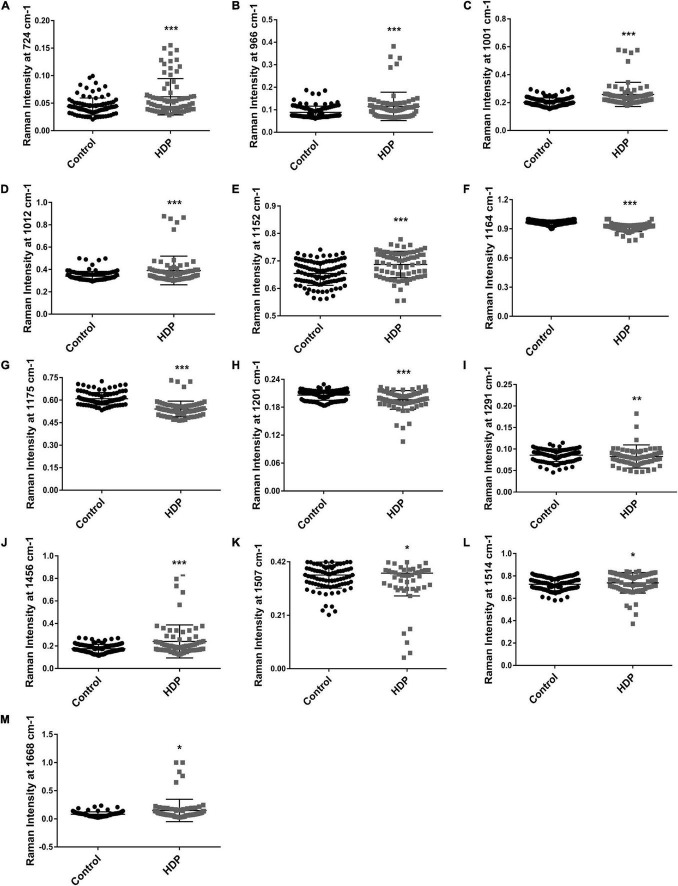
Raman spectral changes from exosome-enriched plasmas. The Raman bands with most variance were further analyzed. Raman intensity from the bands: **(A)** 724 cm^–1^; **(B)** 966 cm^–1^; **(C)** 1,001 cm^–1^; **(D)** 1,012 cm^–1^; **(E)** 1,152 cm^–1^; **(F)** 1,164 cm^–1^; **(G)** 1,175 cm^–1^; **(H)** 1,201 cm^–1^; **(I)** 1,291 cm^–1^; **(J)** 1,456 cm^–1^; **(K)** 1,507 cm^–1^; **(L)** 1,514 cm^–1^; **(M)** 1,668 cm^–1^. Bar graphs represent mean values ± SEM. *p < 0.05; **p < 0.01; ***p < 0.001.

The patients with HDP had increased the intensities of the following bands in relation to the control: 724 cm^–1^, that had mixed contributions from acetyl-CoA, CoA, methionine and sphingomyelin (*p* < 0.0001) (Figure 3A); 966 cm^–1^ assigned to DNA backbone and RNA (*p* < 0.001) (Figure 3B); 1,001 cm^–1^ assigned to phenylalanine (*p* < 0.0001) (Figure 3C); 1,012 cm^–1^ assigned to tryptophan (*p* = 0.0002) (Figure 3D); 1,152 cm^–1^ assigned to carotenoids (*p* < 0.0001) (Figure 3E); 1,456 cm^–1^ assigned to proteins and phospholipids (*p* < 0.0004) (Figure 3F); 1,507 cm^–1^ assigned to myristic acid (*p* = 0.0185) (Figure 3G); 1,514 cm^–1^ assigned to carotenoids (*p* = 0.0177) (Figure 3H); and the 1,668 cm^–1^ assigned to amide I (β-sheet) (*p* < 0.0236) (Figure 3I). Moreover, the patients with HDP also had decreased bands in relation to the control, which were: 1,164 cm^–1^ assigned to tyrosine and arginine (*p* < 0.0001) (Figure 3J); 1,175 cm^–1^ assigned to tyrosine, leucine and phenylalanine (*p* < 0.0001) (Figure 3K); 1,201 cm^–1^ assigned to tyrosine (*p* = 0.0002) (Figure 3L); and 1,291 cm^–1^ assigned to amide III (α-helix) (*p* = 0.0084) (Figure 3M).

### Analysis of ^1^H NMR Spectra From Plasma Metabolites

**FIGURE 4:**
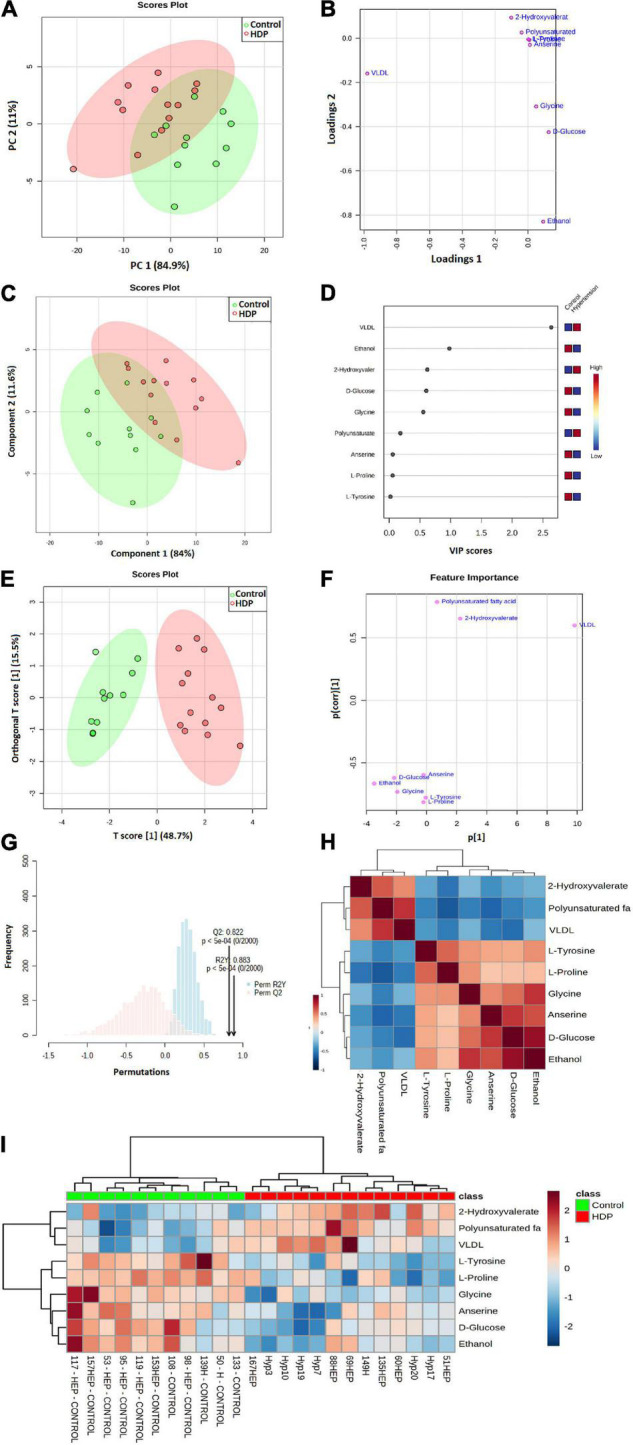
^1^H NMR metabolism analysis from exosome-enriched plasmas. **(A)** Score plot, from unsupervised PCA, with the first two principal components from PCA of exosome-enriched plasma metabolites. Each axis indicates the percentage of total variability explained by the component; **(B)** PC loadings; **(C)** PLS-DA score plot with the first two principal components from PCA of exosome-enriched plasma metabolites; **(D)** PLS VIP; **(E)** The model OPLS-DA shows the predictive component vs. orthogonal component; **(F)** Feature importance; **(G)** Permutation tests (*n* = 2,000); **(H)** Correlations heatmap; **(I)** Clustering analysis and heatmap visualization.

Our ^1^H NMR PCA score plots of the metabolic profiles did not show a clear separation into two separate clusters, as they partially overlapped (Figure 4A). The score plot was in the Hoteling T^2^ ellipse with 95% confidence. The first two PC loadings explained 95.9% of the variance of the original data set, with PC1 describing 84.9% and PC2 describing 11% (Figure 4A). The main metabolites described in PC1 and PC2 were D-glucose, ethanol, L-proline, L-tyrosine, glycine, anserine, 2-hydroxyvalerate, polyunsaturated fatty acids, and very-low-density lipoprotein (VLDL) (Figure 4B). In addition, we employed the PLS-DA, a statistical method which by emphasizing the differences between groups while minimizing the differences within the group, better discerns the overall characteristics and variation of multidimensional data. The first two variables of the PLS-DA model cumulatively accounted for 95.6% of the total variance, and similarly to PCA, the clusters partially overlapped (Figure 4C). Based on PLS-DA, ANOVA, and fold changes, the variable importance in projection (VIP) score revealed multiple significant alterations, and the VLDL increase in patients with HDP was the most important variable (Figure 4D). The patients with HDP had higher levels of VLDL, 2-hydroxyvalerate, and polyunsaturated fatty acids, whereas had lower levels of D-glucose, ethanol, L-proline, L-tyrosine, glycine, and anserine (Figure 4D).

We also employed OPLS-DA, as it has the ability to separate predictive from non-predictive (orthogonal) variation, thus, representing a powerful extension to PLS-DA. In OPLS-DA, both groups managed to be completely separated into two defined clusters, with VLDL the most important variable (Figures 4E,F). Cross-validation showed a parameter R2Y value of 0.883 and Q2 value of 0.822 (2,000 permutations, *p* < 0.0005) (Figure 4G), defining that ^1^H NMR OPLS-DA has the ability to assign an unknown sample to either the control or HDP groups at a predictive precision of 88.3%.

The correlations plot showed positive correlations between D-glucose, ethanol, L-proline, L-tyrosine, glycine, and anserine, all higher in the control group, whilst also positive correlations were found between VLDL, 2-hydroxyvalerate, and polyunsaturated fatty acids, higher in the HDP group (Figure 4H). In addition, the clustering analysis and heatmap visualization of both groups showed how similar samples amid control or HDP group are, and the main differences between the analyzed groups (Figure 4I), also confirming what was found in correlations plot and OPLS-DA.

**FIGURE 5:**
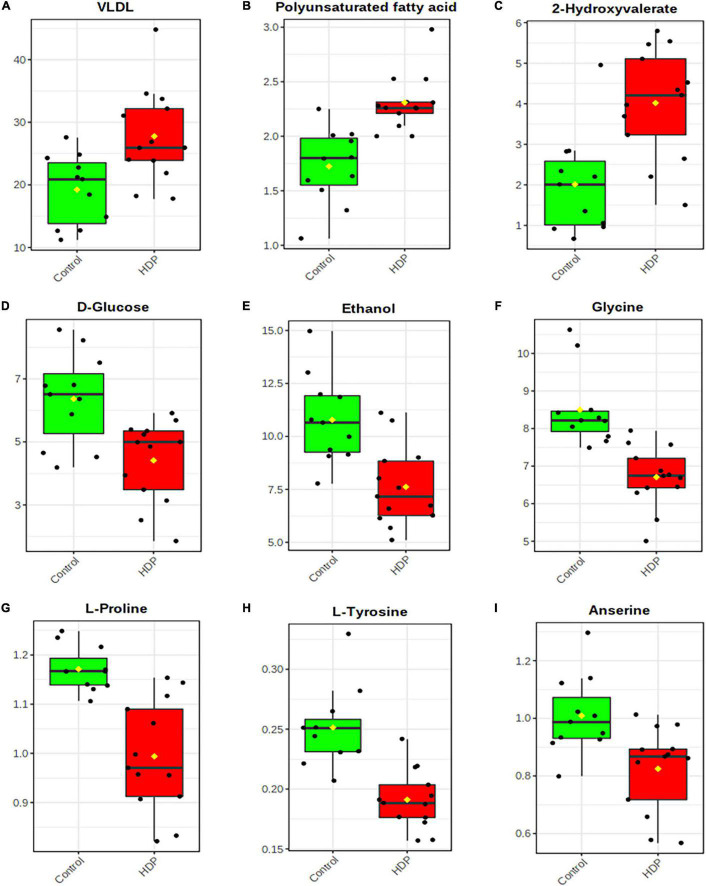
Exosome-enriched plasma metabolite analysis. Box-and-whisker plots of relative concentrations for some significantly altered metabolites (*p* < 0.01): **(A)** VLDL; **(B)** Polyunsaturated fatty acids; **(C)** 2-Hydroxyvalerate; **(D)** D-Glucose; **(E)** Ethanol; **(F)** Glycine; **(G)** L-Proline; **(H)** L-Tyrosine; **(I)** Anserine. Y-axes are represented as relative units and medians are indicated by horizontal lines within each box.

The relative concentrations, calculated by normalization of the molar concentration of each metabolite to the total molar concentration of all metabolites for each sample in the two group, were compared using box-and-whisker plots. As such, the data obtained demonstrated that the HDP group showed had higher levels of VLDL (*p* = 0.00004, Figure 5A), polyunsaturated fatty acids (*p* = 0.00009, Figure 5B) and 2-hydroxyvalerate (*p* = 0.00104, Figure 5C), whilst lower levels of D-glucose (*p* = 0.00215, Figure 5D), ethanol (*p* = 0.00068, Figure 5E), glycine (*p* = 0.00008, Figure 5F), L-proline (*p* = 0.00006, Figure 5G), L-tyrosine (*p* = 0.00004, Figure 5H) and anserine (*p* = 0.00454, Figure 5I), also confirming the other analysis previously performed.

### Predictive Diagnostics Model Building Using ^1^H NMR Spectra From Plasma Metabolites

**FIGURE 6:**
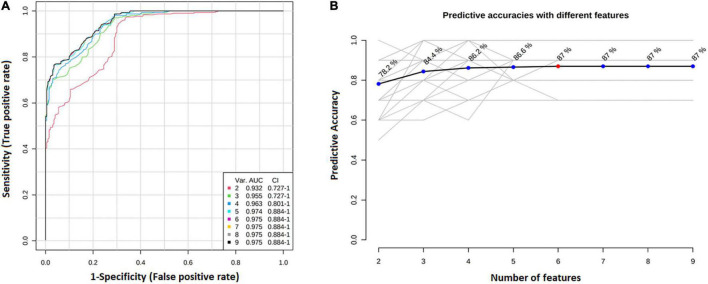
Diagnosis predictive model with all metabolites *via* logistic regression analysis. **(A)** ROC curve for building a diagnostic and predictive model with all metabolites *via* logistic regression analysis. Area Under the Receiver Operating Characteristics (AUROC) was performed on a multivariate algorithm partial least squares-discriminant analysis (PLS-DA). It used the classification method PLS-DA built-in for calculating accuracy, sensitivity, and specificity. The sensitivity and specificity were summarized using the area under the curve (AUC); **(B)** The best predictive accuracy was 87%.

We have performed ROC analysis for building a diagnostic and predictive model with all metabolites *via* logistic regression analysis (Figure 6A). Area Under the Receiver Operating Characteristics (AUROC) was performed on a multivariate algorithm partial least squares-discriminant analysis (PLS-DA). It used the classification method PLS-DA built-in for calculating accuracy, sensitivity, and specificity. The sensitivity and specificity were summarized using the area under the curve (AUC) (Chong et al., 2019). The AUC evaluated the performance of the designed model in the training set. Figure 5A also shows that the model built with all metabolites demonstrated a discriminator AUC > 95%, which reinforced the capacity of metabolite patterns to distinguish between groups. In addition, better predictive accuracy was 87% (Figure 6B).

## Discussion

The establishment and maintenance of a healthy pregnancy depend on intensive maternal-fetal communication. As a major player in homeostasis control, the placenta modulates maternal physiology *via* cell-to-cell communication, facilitated by an increase in the body plasma volume during gestation (18–29% between 14 and 27 weeks). Remarkably, fluctuations in plasma volume, especially the decrease of overall volume during pregnancy, can be indicative of gestational disorders, such as HDP (Aguree and Gernand, 2019; Czernek and Düchler, 2020). Extensively present in the peripheral blood plasma during early gestation, small extracellular vesicles (exosomes) are well described as important mediators of maternal-fetal communication. The wide distribution, the heterogeneous origins of plasma exosomes, and their ability to deliver proteins, metabolites, nucleic acids, and lipids, make the peripheral blood plasma an excellent source for biomarkers research as well as diagnosis and prognosis of gestational diseases (Palviainen et al., 2020; Matsubara et al., 2021). Considering that, during gestation, the concentration and cargo of exosomes, from both maternal and placental cellular origins, provides an overview of the gestational physiological status and their effects on maternal-fetal cell behavior. More importantly, maternal cellular response to placental exosomes creates a temporal mechanism of pregnancy and homeostasis control, that can be related to specific pregnancy-derived exosome populations on normal or pathological gestations (Salomon et al., 2017; Sadovsky et al., 2020).

Despite differential centrifugation being the more affordable method for exosome isolation, it still requires an ultracentrifugation apparatus and the generation of an exosome pellet that can be resuspended. Thus, minimizing the inclusion of plasma protein aggregates during biomarkers research. Nevertheless, it is still feasible to use low-speed centrifugation to remove cell and membrane debris, apoptotic bodies (≥1,000 nm) and large extracellular vesicles 150–1,000 nm microvesicles) from plasma, resulting in an exosome enriched supernatant (Webber and Clayton, 2013; Lobb et al., 2015; Coumans et al., 2017; Turchinovich et al., 2019). Nevertheless, one limitation of our study was to not use normalized samples for exosomes number, and the results may just reflect differences in numbers of isolated exosomes, and not reflect differences in the cargo of the isolated exosomes. To overcome this limitation, we employed NTA analysis and demonstrated that despite the increase in overall particle concentration on HDP samples when compared with controls, the size distribution and concentration of particles between 50 and 150 nm (the vast majority of particles) were unchanged amid the samples. This could relate to differences observed on Raman spectra between HDP and control, as a result of altered exosome cargo and increased exosome concentration. Small extracellular vesicles are generally produced and addressed to a specific target cell. In this context, the increase of particle count on HDP samples could be a possible cause of some altered bands observed in Raman spectroscopy. As an address vesicle, exosomes can also reflect a change of physiological signaling between the mother and the placenta, not only by producing more vesicles with consequent more cargo but also using them as a mechanism of reducing the disposability of certain cargo due to HDP.

Additionally, novel methods on HDP screening, prognosis, and diagnosis are needed, and not only exosomes but Raman spectroscopy and ^1^H NMR have also been proposed to provide better alternatives suited for implementation in low resource settings (Ghoochani et al., 2018; Richards et al., 2021). Regarding Raman spectroscopy, some band intensities were described to be changed in preeclamptic placentas in comparison to healthy placentas, mainly related to tryptophan, tyrosine, and amide I, assigning the amide I difference to protein structural disorders. Also, they found unusual spectra for phenylalanine and tryptophan, which correlated to oxidative modifications that are common in preeclampsia (Chen et al., 2014). In preeclamptic serum, bands related to proteins and amino acids, such as glycine, glutamine, valine, leucine, phenylalanine, tyrosine, and histidine were altered in comparison to control (Başar et al., 2012). Interestingly, we found several similar modifications in HDP samples, which had altered levels of bands related to phenylalanine (1,001 cm^–1^), tryptophan (1,012 cm^–1^), proteins and phospholipids (1,456 cm^–1^), amide I (β-sheet) (1,668 cm^–1^), tyrosine and arginine (1,164 cm^–1^); tyrosine, leucine, and phenylalanine (1,175 cm^–1^); tyrosine (1,201 cm^–1^) and amide III (α-helix) (1,291 cm^–1^).

The addition of plasma solutes and solvents during sample analysis brings more variables to be accessed, without overcrowding the number of variables to be analyzed. Increased levels of plasma lipoproteins, especially those carrying non-coding microRNAs, can contribute to cellular signaling and have the potential to mediate the crosstalk within the cardiovascular system (Li et al., 2018). It is known that the biosynthesis of fatty acids and cholesterols uses large amounts of energy, in turn requiring a large amount of acetyl-CoA, ATP, and oxygen, whereas hypoxia-derived exosomes tend to promote lipid accumulation (Wang et al., 2020). As such, the altered levels of acetyl-CoA, CoA, sphingomyelin, and phospholipids on HPD may reflect the exacerbated production of membrane components and carriers, resulting in the accumulation of fatty acids and the dysregulation of exosome communication leading to poor vascularization and hypertension. Moreover, the increased levels of phospholipids (EVs membrane compound) and sphingomyelin (placental exosome associate protein) direct to placental exosome cargo and composition during gestation (Burkova et al., 2021). Myristic acid also found to have altered band distribution in our analysis, is a long-chain saturated fatty acid, abundant in milk and derivatives (Verruck et al., 2019), and a known cargo of exosomes (Subra et al., 2010). It can be associated with an increase in exosome transportation of myristic acid and the myristic acid concentration is thought to be related to hypertensive disease (Li et al., 2020). In addition, it was described that reduced myristic acid levels are associated with the onset of preeclampsia (Villa et al., 2009; Paik et al., 2017). Therefore, the ability of exosomes to transport myristic acid could be a major regulation pathway on the onset of HDP *via* unbalanced myristic acid delivery.

Altered levels of carotenoids are often associated with diseases or loss of homeostasis, such as preneoplastic changes and cancer, although other studies show that carotenoids, in association with other components, have a protective effect against some chronic diseases (hypertension, diabetes, hypercholesterolemia) (Gomes, 2007). In the study of Palan et al. (2001), the levels of four carotenoids (α-carotene, β-carotene, lycopene, and canthaxanthin) were compared in placental tissue, maternal serum, umbilical cord blood, and it was observed that serum β-carotene and lycopene levels were significantly decreased in preeclampsia. In contrast, our results suggest a possible increase in carotenoid levels in HDP compared to controls, leaving the question of whether the reduction is a result of HDP pathophysiology or a possible response.

Our analyses of enriched plasma demonstrated differences between overall sample composition and different metabolites, as observed in the ^1^H-NMR results described herein. This technique had the ability to assign an unknown sample to either control or HDP groups at a predictive precision of 88.3%, however, further studies need to be conducted to assess if it would be possible to predict HDP prior to the current clinical diagnosis, around 20 weeks of gestation. A set of metabolites was found to be altered in HDP. Notably, increased VLDL in HDP may reflex the presence of 30–85 nm VLDL particles dissolved in the plasma after differential centrifugation (Li et al., 2018). VLDL was also described as an important molecule on HDP pathogenesis (Bawah et al., 2020), and it is an independent contributor to hypertension associated with hyperhomocysteneimia, being considered a biomarker for diagnosis and prognosis (Chen et al., 2021). In addition, VLDL can be metabolized into low-density lipoproteins (LDL) that are transformed in the peripherical tissues in unsaturated fatty acids, which increase cholesterogenesis and peroxisome proliferator-activated receptor (PPAR) family activation, also increasing adipokines and cytokines release, lipid metabolism, gluconeogenesis, and inflammation, thought to be important in HDP pathogenesis (Gutaj et al., 2020). Polyunsaturated fatty acids (PUFA) were also found to be increased in patients with HDP. PUFAs are mainly localized in cytoplasmic membranes and are degraded for energy generation through the citric acid cycle and ketogenesis. Despite the known beneficial effects of omega-6 and omega-3, in patients with HDP, long-chain polyunsaturated fatty acids are thought to increase the risk of preeclampsia and preterm birth (Irwinda et al., 2021). Furthermore, 2-hydroxyvalerate was also noted to be increased in patients with HDP. Interestingly, elevated levels of this fatty acid were also found in placentas from patients with early-onset preeclampsia (Kawasaki et al., 2019).

Several metabolites were found to be reduced in HDP, such as anserine, glycine, L-tyrosine, L-proline, ethanol, and D-glucose. Anserine is a dipeptide containing 3-methylhistidine and β-alanine, being described to have antioxidative and neuroprotective functions (Ding et al., 2018). In anesthetized rats, the anserine injection has a dose-dependent effect on increasing blood pressure (Tanida et al., 2015). Nevertheless, an increase in dietary uptake of anserine is thought to be beneficial for preventing and treating obesity and cardiovascular dysfunctions amid other diseases (Wu et al., 2020). Still, more studies are necessary to assess anserine functions during pregnancy and the effects of its reduction in patients with HDP. Glycine is thought to reduce blood pressure through radical oxidative stress reduction and nitric oxide generation (El-Hafidi et al., 2006). Additionally, *in vivo* studies showed that the increased systolic blood pressure in rats induced by a maternal low-protein diet was reversed by dietary supplementation with glycine (Jackson et al., 2002), which also elicited a dose-dependent fall in mean arterial pressure in normotensive WKY rats (Mishra et al., 2008). Two amino acids were found at reduced levels in patients with HDP in this study. L-Tyrosine reduction is linked to increased risk for insulin resistance and gestational diabetes (Park et al., 2015). Inversely, in ^1^H NMR studies with preeclamptic patients, L-Tyrosine was increased (Turner et al., 2008), while studies using Raman spectroscopy noted reduced levels (Başar et al., 2012). L-Proline levels were not only reduced in our patients with HDP but were also described to be reduced in patients with hypertension (Leal et al., 2019). The D-glucose reduction observed herein was also observed in other studies, associated with preeclampsia/eclampsia incidence (Pugh et al., 2009), although there are studies that related otherwise (Negrato et al., 2009). However, reduced glucose levels are also known to increase peripheral systolic blood pressure (Feldman-Billard et al., 2010; Frier et al., 2011). Although we have excluded the participants that answered to drink alcohol in the anamnesis, it seems that many pregnant women from our study had high levels of ethanol in their plasma, being even elevated in the control group. We believe that ethanol reduction in HDP exosome-enriched plasma samples might be associated with the women knowing their increased risk condition, therefore reducing their alcohol consumption. As we found surprising at first, other studies showed that a high number of Brazilian pregnant women still have high ethanol consumption, from 10 to 40% (Meucci et al., 2017; Martinelli et al., 2019).

In summary, the current study presented exosome enriched plasma as a non-invasive, more accessible technique that could not only lead to biomarkers discovery but also could be used for early diagnosis and prognosis of HDP, particularly in Latin American countries. In particular, the ability of ^1^H NMR OPLS-DA to predict HDP in samples with 88.3% precision, and the logistic regression analysis to build a predictive model with 87% accuracy, strongly suggested that ^1^H NMR is a reliable tool that could be applied on early non-invasive diagnostic method for HDP. Despite these important findings, further investigations in early-stage pregnancies (before 20 weeks) are pivotal to assess if the noted changes in this study are a result of the development of HDP or a response to its progression. Finally, analyzing exosome-enriched plasma samples with Raman Spectroscopy and ^1^H NMR for the investigation of HDP appears to be a powerful tool capable of detecting small changes in biochemical signatures with great sensitivity and specificity that could indeed provide a better approach to HDP early diagnosis and management.

## Data Availability Statement

The raw data supporting the conclusions of this article will be made available by the authors, without undue reservation.

## Ethics Statement

The studies involving human participants were reviewed and approved by subjects were selected at the High-Risk Outpatient Clinic of the Darcy Vargas Maternity, Joinville, Santa Catarina, Brazil, from July to December 2019. Study was approved at the Ethics Committee (3.276.544/2019). The patients/participants provided their written informed consent to participate in this study.

## Author Contributions

SP was responsible for patient data and samples collection and their proper storage. RW was responsible for the project proposal and helped in manuscript writing. MS and EMP helped with patient inclusion and anamnesis. AM, ECS, and EJS were responsible for Raman spectroscopy (RS) measures and statistical analysis. JSU and TA were responsible for ^1^H-NMR measures, spectra interpretations, and statistical analysis. EB and JU were responsible for manuscript revision. JS was responsible for project administration and general supervision. AB was responsible for RS assignments, organizing results and data analysis, and manuscript writing. All authors contributed to the article and approved the submitted version.

## Conflict of Interest

The authors declare that the research was conducted in the absence of any commercial or financial relationships that could be construed as a potential conflict of interest.

## Publisher’s Note

All claims expressed in this article are solely those of the authors and do not necessarily represent those of their affiliated organizations, or those of the publisher, the editors and the reviewers. Any product that may be evaluated in this article, or claim that may be made by its manufacturer, is not guaranteed or endorsed by the publisher.

## Footnotes

1
http://cran.rproject.org/
